# A case of atopic dermatitis caused by *Ascaris lumbricoides* infection

**DOI:** 10.1186/s12948-018-0088-5

**Published:** 2018-04-10

**Authors:** Rosanna Qualizza, Laura M Losappio, Fabiana Furci

**Affiliations:** 1 Allergy Service, ASST Nord Milano, Milan, Italy; 2 Allergy Department, Niguarda Ca’ Granda Hospital, Milan, Italy; 3 School and Operative Unit of Allergy and Clinical Immunology Department of Clinical and Experimental Medicine, University of Messina, Messina, Italy

## Abstract

**Background:**

Parasite infections stimulate total and specific IgE production that, in the case of *Toxocara canis* infection, corresponds to chronic allergic symptoms. There may also be other infections which have similar symptoms, such as *Ascaris lumbricoides* infection. *Ascaris lumbricoides* is a large nematode that causes abdominal pain, nausea, vomiting, bloating, anorexia and intermittent diarrhoea. Patients with ascaridiasis and high IgE levels may also have allergy-like symptoms such as asthma, urticaria and atopic dermatitis.

**Case presentation:**

We report a case of atopic dermatitis caused by Ascaris lumbricoides which shows the important role of parasitic infection in patients with long-lasting dermatitis. The patient was a 12-year old female suffering since early infancy from atopic dermatitis and asthma. She was treated for dermatitis with oral bethametasone and topical pimecrolimus with little benefit. After two cycles of mebendazole therapy, the patient showed progressive improvement of symptoms.

**Conclusions:**

In patients with dermatitis, Ascaris lumbricoides infection should be not excluded: adequate anthelmintic treatment may result in complete regression from the disease.

## Background

In western countries, it is well known that allergic disease prevalence is increasing: this regards respiratory, cutaneous and food allergies [1]. Genetic predisposition, the interaction between genome and environment and the so-called hygiene hypothesis are the mechanisms that seem to be involved. Nevertheless, other aspects need to be studied in depth; one of these is the role of IgE title and factors that can determine its increase. Helminth infections can stimulate total and specific IgE production. It has been described that *Toxocara canis* can induce chronic allergic symptoms [2] and it is thought that other helminth infections can act similarly as a trigger or can maintain an inflammatory status. There is growing international interest in the study of the relationship between helminth infections and allergic diseases. Ascaridiasis is a common intestinal infestation caused by nematodes of the genus ascaridia. *Ascaris lumbricoides* is one of the main common causes of human parasitic infection. Patients with ascaridiasis are generally asymptomatic and may present with symptoms of abdominal pain, nausea, vomiting, bloating, anorexia and intermittent diarrhoea [3]. Patients with ascaridiasis and high IgE levels may have allergy-like symptoms such as asthma, urticaria and atopic dermatitis. This study was aimed at evaluating changes in total and Ascaris-specific IgE levels, as well as in symptoms, following anti-helminthic therapy in patients with atopic dermatitis resistant to standard anti-allergic treatment.

## Case report

**Fig. 1:**
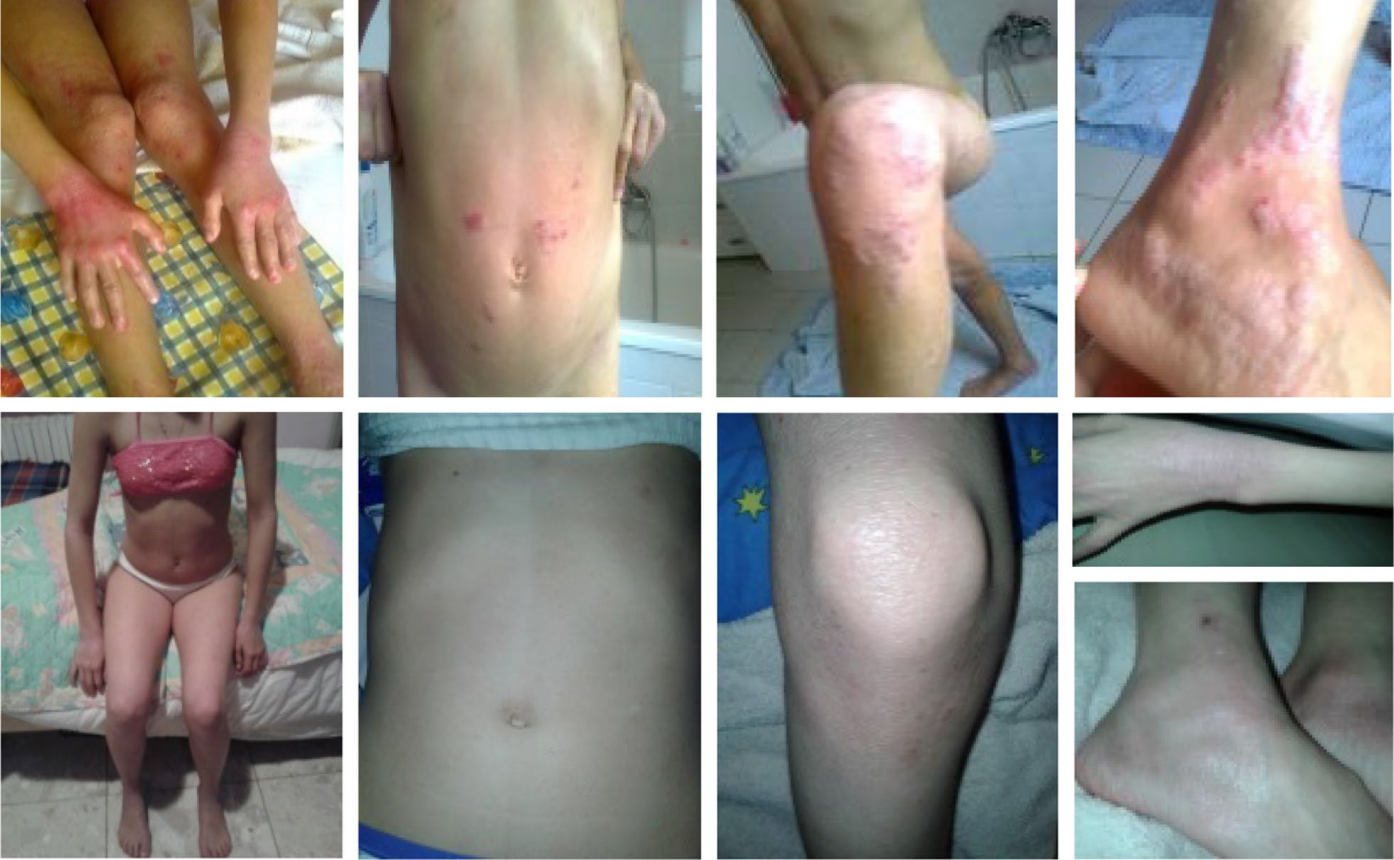
Clinical pictures before and after treatment

The patient was a 12-year old female suffering since early infancy from atopic dermatitis and asthma. Both skin and respiratory symptoms were perennial, with worsening in spring and autumn. Allergy testing, performed at the age of 18 months, resulted positive to *Dermatophagoides pteronyssinus* and *farinae*. In addition, tomato, hen’s egg and cow milk were positive to skin prick tests. Following environmental measures to reduce house dust mite exposure and the elimination of tomato, egg and milk from the diet, there was an improvement of the patient’s asthma condition but not in atopic dermatitis. At 3 and 6 years of age, there was a worsening of dermatitis with modest response to topical corticosteroids, while asthma was no longer present. A further worsening of atopic dermatitis occurred at 9 years of age, which was treated with oral bethametasone and topical pimecrolimus. In September 2014, the patient was referred to our Unit; we found peripheral eosinophilia of 14.4% and, suspecting parasitic infections, we evaluated specific IgE for *A. lumbricoides*, which had a value of 32.50 kU/L. Anthelmintic therapy was prescribed using mebendazole (one 100 mg 1 tablet b.i.d. for 3 days), repeated after 20 and 50 days. Table 1 shows patient data. One month after the first two cycles of therapy, the patient showed progressive improvement of symptoms (Fig. 1), and eosinophilia was 12%. Six months after the end of therapy, the skin was free from dermatitis and a further decrease was observed for eosinophilia (11.20%) and Ascaris-specific IgE (23.90 kU/L).

**Table 1:** Patient data

Skin tests (mm)	Specific IgE levels (kU/L)	Parasitological stools examination	Eosinophils	Total IgE levels (kU/L)			
At 12 years				At 1 year	At 3 years	At 5 years	At 12 years
*Der. pt.* = 8 *Der. f. *= 8 Tomato = 4 Milk = 3.5	*Der. Pt *> 100 *Der. f. *> 100 Tomato = 5.2 Milk = 1.2	Positive to oxyuren	14.4%	355	3253	3356	8324

*Der. pt.*, *Dermatophagoides pteronyssinus*; *Der. f.*, *Dermatophagoides farinae*

## Conclusions

The hygiene hypothesis assumes the presence of an inverse relationship between infections during childhood and the development of atopic disorders that is accompanied by a shift from Th2 response to a Th1 response [4–9]. Clinical and epidemiologic studies have shown that the increasing prevalence of allergic disorders such as asthma, allergic rhinitis, food allergy, eczema and allergic conjunctivitis has an inverse relationship to parasitic infection [10–14]. In several helminthic diseases, IgE is involved in protection of the host against the parasitic agent, consistent with the hypothesis that parasitic infection competes for the IgE-Th2 lymphocyte–eosinophil response [15]. A study highlighted the concept that common domains may exist between species that stimulate the Th2 pathway response (parasites and common allergens), examining some common conserved domains (amino acid sequences) [16]. Thus, helminths are modulators of the host immune system, and infections with these parasites have been associated with protection against allergies and autoimmune disease [17]. However, another study observed a positive association between number of helminth infections and peripheral blood eosinophilia, elevated total IgE, spontaneous production of IL-10 and helminth antigen-stimulated production of Th2 cytokines [18]. An increasing number of helminth infections induces a dose–response effect on allergic inflammatory markers and thus allows us to link these infections to allergic diseases, such as atopic dermatitis. Moreover, the study cited above shows the role of helminth infections on Th2 immune response (e.g. the production of Th2 cytokines by peripheral blood leukocytes stimulated with helminth antigens and the peripheral blood eosinophilia) and on the increase in stimulated IL-10 production which could play a role in the suppression of immediate hypersensitivity reactions in the skin. The present case shows that the role of infection from *A. lumbricoides* in patients with long-lasting dermatitis should not be overlooked. Suitable anti-helminthic treatment may result in complete recovery from the disease. Further studies are needed to understand the molecular mechanisms by which this immune modulation occurs, to explain the complex relationship between ascariasis and susceptibility to childhood atopic dermatitis.
